# Phytotoxic Potential and Biological Activity of Three Synthetic Coumarin Derivatives as New Natural-Like Herbicides

**DOI:** 10.3390/molecules201017883

**Published:** 2015-09-29

**Authors:** Fabrizio Araniti, Raffaella Mancuso, Antonio Lupini, Salvatore V. Giofrè, Francesco Sunseri, Bartolo Gabriele, Maria Rosa Abenavoli

**Affiliations:** 1 Dipartimento AGRARIA, Università Mediterranea di Reggio Calabria, Reggio Calabria 89124, Italy; E-Mails: fabrizio.araniti@unirc.it (F.A.); antonio.lupini@unirc.it (A.L.); francesco.sunseri@unirc.it (F.S.); 2 Laboratory of Industrial and Synthetic Organic Chemistry (LISOC), Dipartimento di Chimica e Tecnologie Chimiche, Università della Calabria, Via P. Bucci 12/C, Arcavacata di Rende (Cosenza) 87036, Italy; E-Mail: bartolo.gabriele@unical.it; 3 Dipartimento di Scienze del Farmaco e dei Prodotti per la Salute, Università di Messina, Via SS Annunziata, Messina 98168, Italy; E-Mail: salvatorevincenzo.giofre@unime.it

**Keywords:** *Arabidopsis thaliana*, *Amaranthus retroflexus*, *Echinochloa crus-galli*, germination, root morphology, phytotoxicity, natural-like herbicides, coumarins

## Abstract

Coumarin is a natural compound well known for its phytotoxic potential. In the search for new herbicidal compounds to manage weeds, three synthetic derivatives bearing the coumarin scaffold (**1**–**3**), synthesized by a carbonylative organometallic approach, were *in vitro* assayed on germination and root growth of two noxious weeds, *Amaranthus retroflexus* and *Echinochloa crus-galli*. Moreover, the synthetic coumarins **1**–**3** were also *in vitro* assayed on seedlings growth of the model species *Arabidopsis thaliana* to identify the possible physiological targets. All molecules strongly affected seed germination and root growth of both weeds. Interestingly, the effects of synthetic coumarins on weed germination were higher than template natural coumarin, pointing out ED_50_ values ranging from 50–115 µM. Moreover, all synthetic coumarins showed a strong phytotoxic potential on both *Arabidopsis* shoot and root growth, causing a strong reduction in shoot fresh weight (ED_50_ values ≤ 60 µM), accompanied by leaf development and a decrease in pigment content. Furthermore, they caused a strong alteration in root growth (ED_50_ values ≤ 170 µM) and morphology with evident alterations in root tip anatomy. Taken together, our results highlight the promising potential herbicidal activity of these compounds.

## 1. Introduction

Weeds are still the most important pest because they compete with crops for water and nutrient resources reducing yields and quality and, consequently, causing huge economic losses [1,2,3,4]. Although mechanical, chemical, biological and cultural practices for weed management have evolved over the past century, synthetic herbicides currently represent the most common, effective and economical method for their control among all major crops [5,6]. However, concerns for the negative impact of synthetic herbicides on the environment and human health along with the rapid evolution of weeds’ resistance made the search for new herbicides with a new mode of action (MOA) and/or a new weed management paradigm more pressing and stringent [7]. Large efforts should be undertaken to develop alternative methods for weed control, using eco-friendly, cost-effective and bioactive natural or natural-like products. These compounds could be used directly as bio-herbicides or as templates for the production of synthetic herbicides [8]. Although many natural products have been employed to develop different conventional pesticides (e.g., piretroids) and fungicides, few herbicide examples may be provided [9]. Among them, leptospermone, a natural triketone found in the bottlebrush plant extract (*Callistemon citrinus* spp.) [10], is able to repress growth of several weeds [11,12]. This compound was then utilized to synthetize a more potent derivative, mesotrione (Callisto^®^ herbicide), successfully employed as a pre-emergence and post-emergence herbicide. Cinmethylin, a herbicidal analogue of 1,4-cineole, is a moderately effective growth inhibitor used for monocotyledonous weed control [13], whereas pelargonic acid [14], sarmentine [15] and citral [16] are patented natural compounds isolated from plants, known for their high phytotoxic potential against the most common and noxious weeds.

However, there are few natural product-based phytotoxins described in the academic and patent literature. The industry is realizing that many natural compounds could have high potential as *template* for commercially successful herbicides, offering several economic and environmental advantages. 

In the present work, three synthetic coumarin derivatives **1**–**3** were synthesized by PdI_2_/KI-catalyzed dicarbonylation of 2-(1-hydroxyprop-2-ynyl)phenols, and their biological activity, have been evaluated. Coumarins are secondary metabolites containing a 2*H*-1-benzopyran-2-one or benzopyrone moiety [17], largely known for their phytotoxic activity in many plant species. They affect many physiological processes [18] including photosynthesis [19,20,21], nutrient uptake and metabolism [22,23], seed germination [24,25,26] and root growth [27,28,29]. In particular, coumarin, the most simple and representative compound of this class, shows a high phytotoxic potential [30], and its relatively simple chemical structure makes it an excellent *template* for the synthesis of new structural analogues to develop novel natural herbicides.

In this context, the aim of the present paper was: (1) to assess the phytotoxic potential of the synthetic coumarin derivatives **1**–**3** on the noxious weeds, *Amaranthus retroflexus* and *Echinochloa crus-galli*, in order to evaluate their use as promising natural-like herbicides; and (2) to evaluate the phytotoxicity of these molecules on the model species *Arabidopsis thaliana* in order to identify their possible physiological targets.

## 2. Results

### 2.1. Synthesis of 3-[(Methoxycarbonyl)methyl]coumarins **1**–**3**

**Scheme 1:**
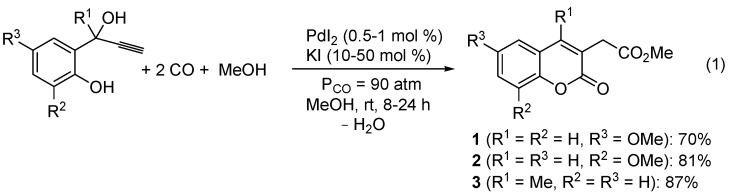
Synthesis of coumarin derivatives **1**–**3** by PdI_2_/KI-catalyzed dicarbonylation of 2-(1-hydroxyprop-2-ynyl)phenols [36].

**Scheme 2:**
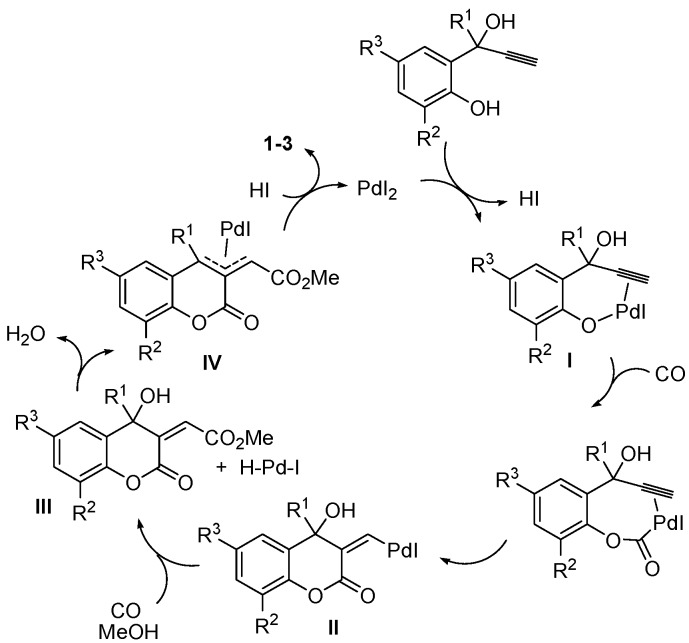
Possible reaction mechanism leading to coumarin derivatives **1**–**3** by PdI_2_/KI-catalyzed dicarbonylation of 2-(1-hydroxyprop-2-ynyl)phenols.

Methyl (6-methoxy-2-oxo-2*H*-chromen-3-yl)acetate (**1**), methyl (8-methoxy-2-oxo-2*H*-chromen-3-yl)acetate (**2**), and methyl (4-methyl-2-oxo-2*H*-chromen-3-yl)acetate (**3**) were synthesized by PdI_2_/KI-catalyzed carbonylation [31,32,33,34,35] of readily available 2-(1-hydroxyprop-2-ynyl)phenols, according to Scheme 1 [36]. Reactions were carried out with 0.5–1 mol % of PdI_2_ in conjunction with 10–50 mol % of KI, at room temperature in MeOH as the solvent and under 90 atm of carbon monoxide (see the Experimental Section for details). A possible pathway leading to the coumarin derivatives involves the formation of a palladium phenate stabilized by triple bond coordination (**I**), followed by CO insertion, intramolecular *syn* insertion of the triple bond and alkoxycarbonylation of the resulting vinylpalladium intermediate (**II**). This leads to the formation of an allylalcoholic intermediate (**III**) and an H-Pd-I species, which, according to a known reactivity, react to give an allypalladium intermediate (**IV**) with elimination of water. Protonolysis of **IV** by HI eventually yields the final coumarin product with regeneration of PdI_2_ (Scheme 2; anionic iodide ligands are omitted for clarity).

### 2.2. Bioassays on Weeds

#### 2.2.1. Weed Germination Bioassay

All the synthetic coumarin derivatives **1**–**3** caused a strong inhibition of all the germination parameters on both weeds (Table 1). This effect was dose-dependent and statistically significant already at the lowest doses (Table 1). In particular, the total germination (G_T_ %) was completely inhibited by 100 µM **1** and 200 µM **2** and **3** in *A. retroflexus*, while the same effect was observed with 100 µM **1** and **2** and 400 µM **3** in *E. crus-galli* (Table 1). In addition, germination speed (S) and speed of accumulated germination (AS) were strongly affected by all molecules in both species (Table 1).

The nonlinear regression fit of data related to G_T_ (%) is characterized by a high statistical significance (*p* < 0.001) in both weeds. In *A. retroflexus*, the comparison of ED_50_ values confirmed the highest phytotoxicity of **1** and **2**, able to cause 50% inhibition of G_T_ at 54 and 58 µM concentrations, respectively, while **3** caused the same effect at 115 µM concentration (Table 1). On the other hand, **2** was the most bioactive molecule (72 µM) against *E. crus-galli*, followed by **1** and **3**, which were both able to cause a 50% inhibition of G_T_ at 105 µM concentration (Table 1).

In a complementary experiment, after each treatment, the un-germinated seeds of both weeds transferred in distilled water were able to recover seed germination ability underlining that the effect of all molecules, at all the concentrations assayed, was reversible.

#### 2.2.2. Root Growth Bioassay

The effects of three synthetic coumarins (**1**–**3**) on root growth of *A. retroflexus* and *E. crus-galli* are reported in Table 1. All the molecules strongly inhibited root growth, and this effect increased along with the increase of molecule concentrations in both weeds (Table 1). The non-linear regression fit of data related to TRL show a high statistical significance (*p* < 0.001), as observed for seed germination. In *A. retroflexus*, **2** showed the most inhibitory effect (105 µM) followed by **3** (126 µM) and **1** (164 µM) (Table 1), whereas in *E. crus-galli* all the molecules assayed were able to cause a 50% of root growth inhibition at ~100 µM concentration (Table 1).

**Table 1:** Effects of the coumarin derivatives **1**–**3** on seed germination (GT %), speed of germination (S), speed of accumulated germination (AS) and Total Root Length (TRL) of *A. retroflexus and E. crus-galli*.

Physiological Process	*A. retroflexus*			*E. crus-galli*		
G_T_ (%)	1	2	3	1	2	3
0 µM	100 (0.0) ^a^	100 (0.0) ^a^	100 (0.0) ^a^	100 (0.0) ^a^	100 (0.0) ^a^	100 (0.0) ^a^
50 µM	81.8 (3.6) ^b^	66 (5.1) ^b^	94 (4.1) ^a,b^	87.8 (4.9) ^b^	59.5 (7.9) ^b^	92 (4.9) ^ab^
100 µM	0 (0.0) ^c^	8 (3.7) ^c^	84 (3.8) ^b^	54 (3.0) ^c^	46.5 (6.3) ^b^	54 (3.0) ^b^
200 µM	0 (0.0) ^c^	0 (0.0) ^d^	0 (0.0) ^c^	0 (0.0) ^d^	0 (0.0) ^c^	10.2 (3.2) ^c^
400 µM	0 (0.0) ^c^	0 (0.0) ^d^	0 (0.0) ^c^	0 (0.0) ^d^	0 (0.0) ^c^	0 (0.0) ^d^
800 µM	0 (0.0) ^c^	0 (0.0) ^d^	0 (0.0) ^c^	0 (0.0) ^d^	0 (0.0) ^c^	0 (0.0) ^d^
ED_50_ (µM)	53.8 (1.3) ^a^	58 (1.6) ^a^	115.3 (5.8) ^b^	104.6 (2.4) ^b^	72.3 (7.6) ^a^	104.9 (3.4) ^b^
S						
0 µM	100 (0.0) ^a^	100 (0.0) ^a^	100 (0.0) ^a^	100 (0.0) ^a^	100 (0.0) ^a^	100 (0.0) ^a^
50 µM	42.5 (2.4) ^b^	37.2 (2.4) ^b^	57.3 (2.5) ^b^	65.5 (4.1) ^b^	42.2 (5.1) ^b^	57.5 (3.9) ^b^
100 µM	0 (0.0) ^c^	3.6 (1.8) ^c^	41.9 (1.9) ^b^	35.2 (3.8) ^c^	23.9 (3.4) ^c^	25.8 (1.7) ^c^
200 µM	0 (0.0) ^c^	0 (0.0) ^c^	0 (0.0) ^c^	0 (0.0) ^d^	0 (0.0) ^d^	4.7 (1.5) ^d^
400 µM	0 (0.0) ^c^	0 (0.0) ^c^	0 (0.0) ^c^	0 (0.0) ^d^	0 (0.0) ^d^	0 (0.0) ^d^
800 µM	0 (0.0) ^c^	0 (0.0) ^c^	0 (0.0) ^c^	0 (0.0) ^d^	0 (0.0) ^d^	0 (0.0) ^d^
AS						
0 µM	100 (0.0) ^a^	100 (0.0) ^a^	100 (0.0) ^a^	100 (0.0) ^a^	100 (0.0) ^a^	100 (0.0) ^a^
50 µM	31.1 (1.9) ^b^	29.7 (2.1) ^b^	51.3 (2.2) ^b^	55.5 (4.6) ^b^	38.5 (4.6) ^b^	47.2 (3.4) ^b^
100 µM	0 (0.0) ^c^	2.1(1.0) ^c^	30.1 (1.3) ^c^	24.6 (3.9) ^c^	14.3 (2.4) ^c^	15.2 (1.4) ^c^
200 µM	0 (0.0) ^c^	0 (0.0) ^c^	0 (0.0) ^d^	0 (0.0) ^d^	0 (0.0) ^d^	2.7 (0.9) ^d^
400 µM	0 (0.0) ^c^	0 (0.0) ^c^	0 (0.0) ^d^	0 (0.0) ^d^	0 (0.0) ^d^	0 (0.0) ^d^
800 µM	0 (0.0) ^c^	0 (0.0)	0 (0.0) ^d^	0 (0.0) ^d^	0 (0.0) ^d^	0 (0.0) ^d^
TRL (cm)						
0 µM	100 (0.0) ^a^	100 (0.0) ^a^	100 (0.0) ^a^	100 (0.0) ^a^	100 (0.0) ^a^	100 (0.0) ^a^
50 µM	76.9 (5.0) ^b^	67.1 (2.0) ^b^	83.9 (5.1) ^b^	69.5 (3.9) ^b^	74.9 (11.6) ^b^	75.3 (2.7) ^b^
100 µM	63.5 (1.6) ^c^	47.5 (2.9) ^c^	51.2 (0.6) ^c^	56.8 (5.0) ^c^	47.8 (10.1) ^b^	49.8 (4.5) ^c^
200 µM	46.4 (3.5) ^d^	41.6 (1.0) ^c^	36.8 (0.6) ^d^	25.1 (4.2) ^d^	21.6 (2.6) ^c^	26.3 (1.7) ^d^
400 µM	27 (2.3) ^e^	20.8 (1.6) ^d^	22.6 (1.4) ^e^	9.4 (2.2) ^e^	6.9 (1.1) ^d^	14.2 (2.9) ^e^
800 µM	8.7 (1.3) ^f^	7.1 (2.2) ^e^	7.7 (0.8) ^f^	4.2 (0.7) ^f^	4.7 (0.4) ^e^	4.7 (0.6) ^f^
ED_50_ (µM)	164 (9.4) ^c^	105.8 (8.9) ^a^	126.4 (9.4) ^b^	104 (6.3) ^a^	94.7 (7.4) ^a^	101.8 (4.3) ^a^

Data are expressed as percentage of the control. Different letters (a–f) along the columns, or along the row (ED_50_ parameter), indicate significant differences at *p* < 0.05. Homoscedastic data were analyzed by ANOVA with Tukey’s test, whereas the heteroscedastic ones were analyzed with Tamhane’s T2. Values within the brackets indicated the standard deviation (*N* = 5). All the dose response curves pointed out a significance level of *p* < 0.001.

### 2.3. Bioassays of Arabidopsis thaliana

#### 2.3.1. Effects of the Synthetic Coumarins on Fresh Weight, Leaf Number and Pigments Content

**Figure 1:**
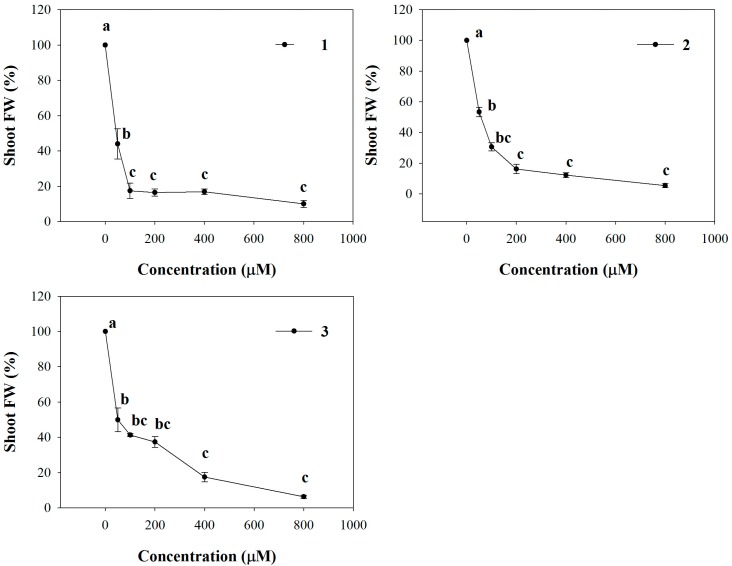
Dose-response curves of shoot fresh weight (SFW) of *A. thaliana* seedlings treated with **1**–**3** for 15 days. Data are expressed as percentage of the control. Different letters (a–c) along the curves indicate significant differences at *p* ≤ 0.05). Homoscedastic data were analyzed by ANOVA with Tukey’s test, whereas the heteroscedastic ones were analyzed with Tamhane’s T2. *N* = 5.

All the molecules significantly affected shoot fresh weight (SFW) of *Arabidopsis* seedlings already at the lowest concentration (50 µM), causing 66%, 46% and 50% of reduction with **1**, **2** and **3** treatments, respectively (Figure 1). Furthermore, this effect was most evident along with increasing of concentrations, reaching almost the complete inhibition at the highest concentration. The non-linear regression fits of the dose-response curves are characterized by a high statistical significance (*p* ≤ 0.001) and all the molecules showed very low ED_50_ values. In particular, comparing the ED_50_ values, **1** seemed to be more harmful showing a higher ED_50_ (26 µM) compared to **2** (53 µM) and **3** (59.3 µM), which did not significantly vary (Table 2).

**Table 2:** ED_50_ (µM) values of shoot fresh weight (SFW), total root length (TRL), number of lateral root (NLR) and root hair density (RHD) of *A. thaliana* estimated by the log-logistic equations in response to different concentrations of **1**–**3**. Data from Figure 1 and Figure 3.

Molecules	ED_50_ (µM)			
	SFW	TRL	NLR	RHD
1	26.01 (0.16) ^a^	111.29 (19.3) ^ab^	42.49 (0.75) ^a^	55.36 (0.28) ^a^
2	53.07 (0.76) ^b^	74.24 (10.1) ^a^	43.56 (0.89) ^a^	56.18 (0.78) ^a^
3	59.27 (0.22) ^b^	173 (18.78) ^b^	43.34 (0.12) ^a^	49.27 (0.46) ^a^

Different letters (a–b) along the columns indicate significant differences at *p* < 0.05. Homoscedastic data were analyzed by ANOVA with Tukey’s test, whereas the heteroscedastic ones were analyzed with Tamhane’s T2. *N* = 5. Values within the brackets indicated the standard deviation (*N* = 5). All the dose-response curves pointed out a significance level of *p* < 0.001.

**Figure 2:**
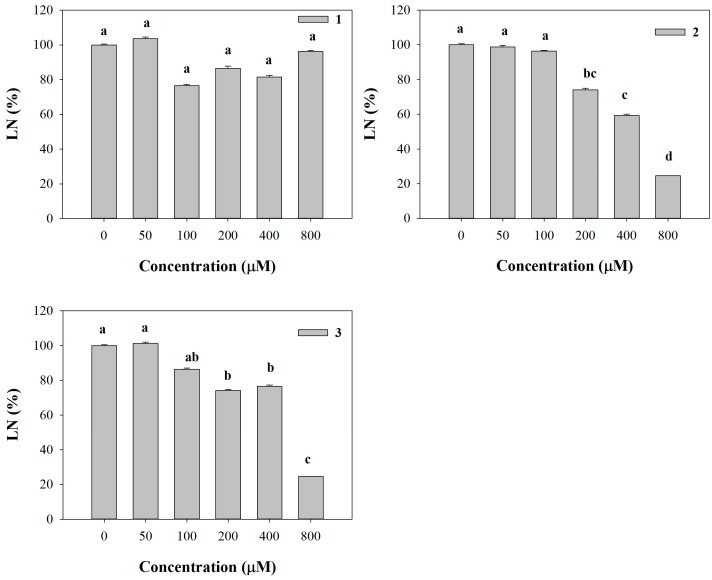
Leaf number (LN) of *A. thaliana* seedlings treated with synthetic coumarins **1**–**3** Data are expressed as percentage of the control. Different letters (a–d) along the bars indicate significant differences at *p* ≤ 0.05). Homoscedastic data were analyzed by ANOVA with Tukey’s test, whereas the heteroscedastic ones were analyzed with Tamhane’s T2. *N* = 5.

**Figure 3:**
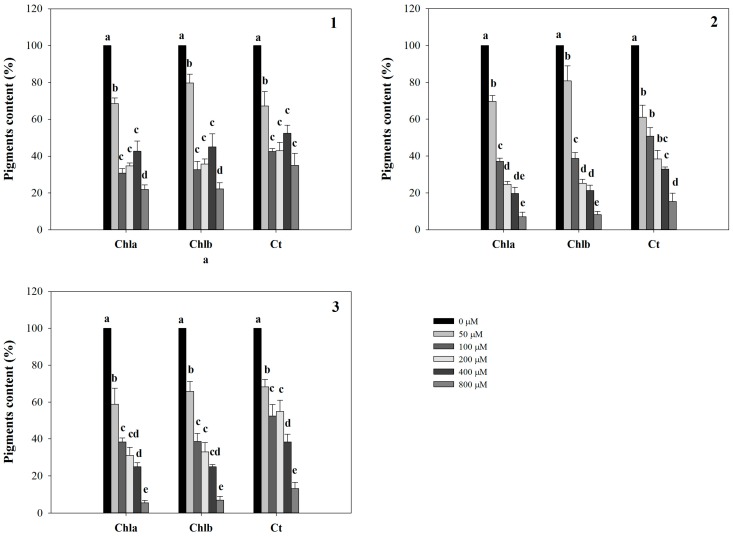
Pigments content in *A. thaliana* shoots treated with synthetic coumarins **1**–**3**. Data are expressed as percentage of the control. Different letters (a–e) along the bars indicate significant differences at *p* ≤ 0.05). Homoscedastic data were analyzed by ANOVA with Tukey’s test, whereas the heteroscedastic ones were analyzed with Tamhane’s T2. *N* = 5.

Conversely, LN was significantly affected only by **2** and **3**, which were able to reduce it already at 200 µM (26% of inhibition), reaching the highest level of inhibition (76%) with both molecules at 800 µM (Figure 2). Furthermore, all the molecules strongly affected leaf area (LA) starting from the lowest concentration (50 µM) (data not shown). Interestingly, similar strong inhibitory effects were observed on pigment content coupled with a chlorotic appearance in *Arabidopsis* leaves treated with the lowest concentration (50 µM). These effects, observed at naked eye, were also confirmed by the experimental data which pointed out a strong dose-dependent reduction in all the pigments quantified (Figure 3).

#### 2.3.2. Root Growth and Morphology

**Figure 4:**
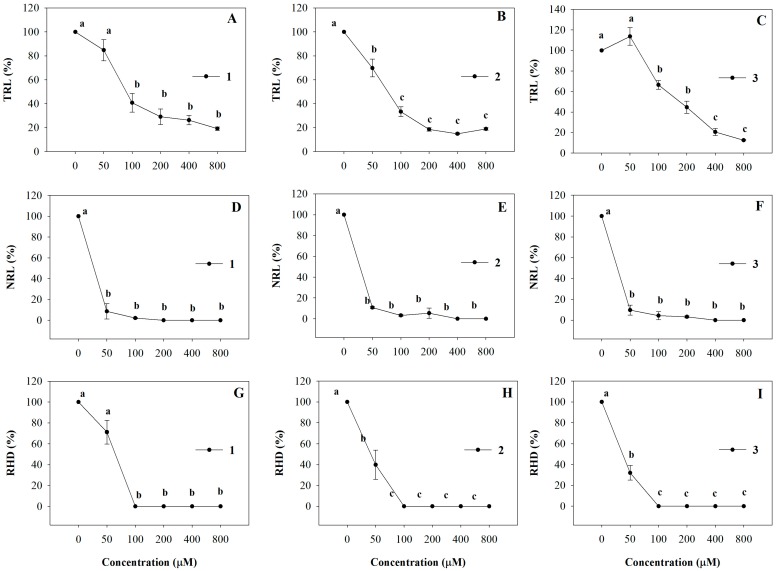
Dose-response curves of root morphology of *A. thaliana* seedlings treated with **1**–**3** for 15 days: (**A**–**C**) Total Root Length (TRL); (**D**–**F**) Lateral Root Number (NLR); (**G**–**I**) Root Hair Density (RHD). Data are expressed as percentage of the control. Different letters (a–c) along the curves indicate significant differences at *p* ≤ 0.05). Homoscedastic data were analyzed by ANOVA with Tukey’s test, whereas the heteroscedastic ones were analyzed with Tamhane’s T2. *N* = 5.

All the molecules strongly affected *A. thaliana* root morphology showing a dose-dependent inhibitory effect on primary root growth. In particular, 100 µM of **1**, **2** and **3** caused 60%, 77% and 44% of TRL inhibition, respectively, and this effect increased as their concentrations increased, reaching 90% of inhibition at the highest concentration (Figure 4A–C). The ED_50_ values were equal to 111, 74 and 173 µM (R^2^ = 0.98) for **1**, **2** and **3**, respectively (Table 2). Interestingly, NLR and RHD root traits were also strongly affected by all synthetic coumarins and the percentage of inhibition showed a similar trend for all the molecules (Figure 4D–I). In detail, *Arabidopsis* seedlings showed 90% of NLR inhibition already at the lowest concentration (50 µM), reaching 100% of inhibition at all higher concentrations (Figure 4D–F). A marked reduction of the RHD was observed already at the lowest concentration (50 µM) which reached a complete inhibition at 100 µM and this effect was confirmed by the ED_50_ values (Figure 4, Table 2).

**Figure 5:**
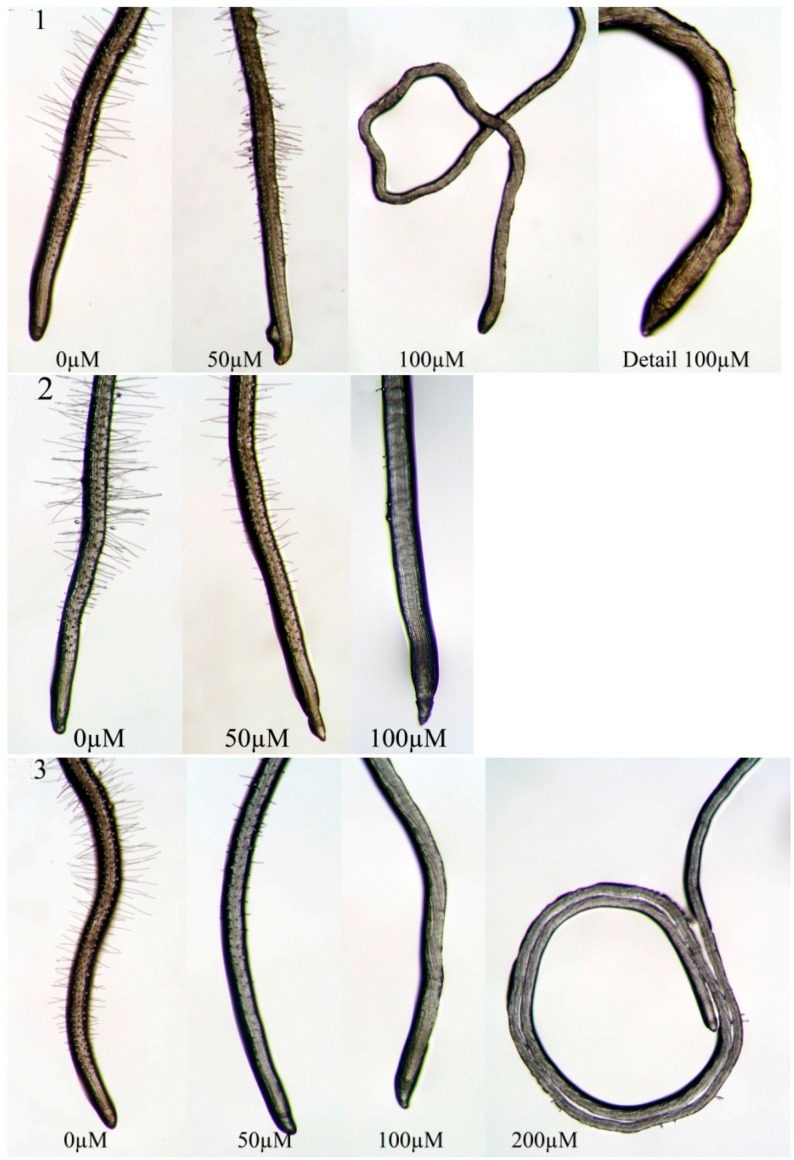
Root apex of *A. thaliana* grown *in vitro* and treated with different concentrations of **1**–**3**. The anticlockwise torsion of the cell files and the absence of root hairs (when treated with molecules **1** and **3**) are evident.

However, root structure and organization were only modified by **1** and **3**, which were able to cause a loss of the gravitropic response already at low concentrations (50 µM) (Figure 5). This effect became evident at 100 µM together with a strong root deformation causing a corkscrew-shape. Moreover, **3** treatment also determined a circumnutation phenomenon (Figure 5).

#### 2.3.3. Number of Mitotic Sites

**Figure 6:**
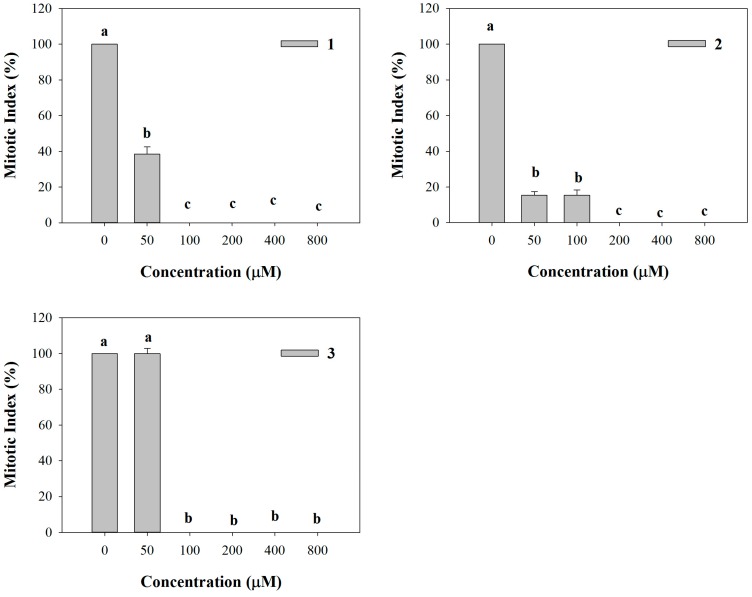
Mitotic sites number evaluated along the primary root of *A. thaliana* seedlings treated with the synthetic coumarins **1**–**3**. Data are expressed as percentage of the control. Different letters (a–c) along the curves indicate significant differences at *p* ≤ 0.05). Homoscedastic data were analyzed by ANOVA with Tukey’s test, whereas the heteroscedastic ones were analyzed with Tamhane’s T2. *N* = 5.

All the synthetic coumarins affected lateral root initiation (Figure 6). In particular, **1** strongly reduced the number of mitotic sites (62% of inhibition) along the primary root, whereas the higher concentrations completely inhibited mitotic sites’ formation (Figure 6). Fifty and 100 µM **2** concentrations already caused 85% of inhibition, reaching a complete inhibition of mitotic sites’ formation at the higher concentrations, as already observed for **1** (Figure 6). Conversely, **3** did not affect mitotic sites’ formation at the lowest concentration (50 µM), whereas at the higher ones it exhibited the same trend of **1** and **2** (Figure 6).

## 3. Discussion

Because of their multifaceted biological activity and broad spectrum of action, coumarins are currently of great interest as a source for new chemical structures with potential utilization in pharmacology, chemical and agronomy fields. Focusing on their potential application in agriculture, the development of new synthetic methods for natural herbicide production starting from the coumarin nucleus as a natural template could provide interesting tools for the development of natural herbicide models. Coumarin, the simplest compound of this class, is well known for its phytotoxicity in germination and growth of many species [24,25,37,38,39], and could represent a promising template for natural herbicides’ production. In this paper, we have evaluated the phytotoxic potential among widespread noxious weeds and *Arabidopsis* model plant of three coumarin derivatives, methyl (6-methoxy-2-oxo-2*H*-chromen-3-yl)acetate (**1**), methyl (8-methoxy-2-oxo-2*H*-chromen-3-yl)acetate (**2**), and methyl (4-methyl-2-oxo-2*H*-chromen-3-yl)acetate (**3**).

Similarly to the natural coumarin template [24,25], all the synthetic molecules exerted a dose-dependent inhibitory effect on seed germination of both noxious weeds. Interestingly, they showed a stronger biological activity compared to coumarin, which is generally considered the most active compound tested among structural analogues [40]. Indeed, 1 mM natural coumarin caused 60% and 40% inhibition in germination of *E. crus-galli* and *A. retroflexus*, respectively [41,42], and more than 60% in wheat seeds’ germination [24], while **1**, **2** and **3** completely inhibited this process at lower concentrations. Similarly, Prabodh *et al.* [43] and Khanh *et al.* [44] observed that higher concentrations of template coumarin (above of 350 µM) were needed to reach 50% inhibition of *Lactuca sativa*, *Lolium perenne* and *E. crus-galli* germination process. Comparable results were also observed by Saleh and Abu El-Soud [45] on wheat germination. Also, other natural coumarins isolated from Meliaceae and Rutaceae, such as psoralen, showed lowest phytotoxic activity on germination process causing the complete inhibition at 1mM concentrations [46]. However, despite their potency, the effects of **1**–**3** were reverted after a careful seed washing of both weeds, allowing them to recover germination in the presence of deionized water, thereafter. This effect, observed also after treatment with natural coumarin [24], suggested that all the molecules did not cause an irreversible germination block. It has been demonstrated that exogenous application of phenols to weeds delayed but did not substantially inhibit germination [41]. The **1**, **2** and **3** treatments also caused a strong dose-dependent inhibitory effect observed on root growth of both weeds. In accordance with coumarin effects on root growth of *E. crus-galli* [42], 100 µM of all the synthetic coumarin derivatives caused 50% inhibition of root growth on both weeds reaching 90% with the highest concentration (800 µM). Conversely, recent results demonstrated that 430 µM (*L. perenne* and *L. sativa*) and 690 µM (*E. crusgalli*) coumarin concentrations were needed to reach 50% inhibition of root growth of these species [43,44]. 

Given their high phytotoxicity on weeds, the effects of these synthetic coumarins were evaluated on shoot and root growth of model species *Arabidopsis thaliana*. As reported by Pennacchio *et al.* [47], this species is sensitive to different allelochemicals and satisfies all of the selection criteria for target species such as fast growth, short life cycle, and mutant availability useful to better identify sites target and mode of action of molecules [48].

All the molecules strongly affected *A. thaliana* growth causing an evident chlorosis, a reduction of leaf number (LN), shoot fresh weight (SFW) and leaf area (LA), together with several alterations in root growth and morphology. In particular, **1**, at all concentrations, reduced SFW without a consistent reduction of leaf number, indicating that this molecule caused a biomass loss without interfering with leaf differentiation. Conversely, **2** and **3**, at concentrations higher than 100 µM, caused a dose dependent LA and LN inhibition which matched SFW responses. Similar results were already observed by Sánchez-Moreiras *et al.* [49] in *Arabidopsis* treated with 2-3*H*-benzoxazolinone (BOA), an allelochemical mainly found in the *Poaceae* [50,51] and largely known for its phytotoxic effect [8,52,53]. Furthermore, similar to natural coumarin, the synthetic coumarin derivatives inhibited in a dose dependent-manner chlorophyll and carotenoids content of *A. thaliana*. Knypl [54,55] showed that coumarin was able to delay the loss of chlorophyll from leaves kept in the dark and to stimulate yellowing in light. Moreover, some natural 4-phenylcoumarins and imperatorin, a furanocoumarin, acted as photophosphorylation uncouplers or energy transfer inhibitors [21,56,57]. Macias *et al.* [58] demonstrated that several coumarins reduced chlorophyll content and photosynthetic activity in spinach chloroplast. Since the chlorophyll content is strictly related to plant biomass production [59], any reduction in leaf pigments would limit net photosynthesis altering the entire plant metabolism. However, if the reduction in chlorophyll content was due to a synthesis inhibition or a chemical-induced degradation was not clarified. Probably, the strong effect induced by these synthetic coumarins on root growth, essential for nutrient uptake, could be indirectly responsible for shortage of some nutrients involved in chlorophyll synthesis. Although natural coumarin increased nitrate uptake in wheat roots [22], many phenolic and synthetic diphenolic compounds markedly reduced net nutrient uptake such as nitrate, ammonium, potassium and phosphorus in different plant species [60,61,62].

All the synthetic molecules strongly affected the root system more than the natural coumarins [45,46] inducing modifications on root morphology and anatomy, such as unaligned cells, gravitropism loss and root hair inhibition. Similar results were observed by several authors in treated roots with oryzalin, taxol and colchicines, affecting microtubule organization [63,64,65], which could also justify root hair loss [66,67]. Furthermore, they caused a strong decrease in the number of lateral roots accompanied by a reduction in mitotic sites’ formation attributable to a biased hormonal balance. Indeed, the formation of lateral roots is dependent on the coordinated action of many factors among which auxin transport and redistribution together with ethylene played a key role [68,69]. These effects were also found in *Arabidopsis* roots after treatment with 3-(methoxycarbonylmethylene)isobenzofuran-1-imines, molecules synthetized from simple substrates by a catalytic carbonylation approach that have showed a very promising potential herbicidal activity [70]. 

## 4. Experimental Section

### 4.1. General Information

Chemicals were purchased from Sigma-Aldrich Italia (Milano, Italy) and were used as such without further purification. Melting points were taken on a Reichert Thermovar apparatus and are uncorrected. ^1^H-NMR and ^13^C-NMR spectra were recorded at 25 °C in CDCl_3_ solutions with a Bruker DPX Avance 300 spectrometer (Bruker Italia, Milano, Italy) operating at 300 MHz and 75 MHz, respectively, with Me_4_Si as internal standard. IR spectra were taken with a JASCO FT-IR 4200 spectrometer (Jasco Europe s.r.l., Cremella, Lecco, Italy). Mass spectra were obtained using a Shimadzu QP-2010 GC-MS apparatus (Shimadzu Italia, Milano, Italy) at 70 eV ionization voltage. Microanalyses were carried out with a Carlo Erba Elemental Analyzer Mod. 1106 (Carlo Erba, Cornaredo, Milano, Italy). All reactions were analyzed by TLC (Merck Italy, Vimodrone, Milano, Italy) on silica gel 60 F254 (Merck Italy) or on neutral alumina (Merck Italy) and by GLC using a Shimadzu GC-2010 gas chromatograph (Shimadzu Italia, Milano, Italy) and capillary columns with polymethylsilicone + 5% polyphenylsilicone as the stationary phase (HP-5). Column chromatography was performed on silica gel 60 (Merck Italy, 70–230 mesh). Evaporation refers to the removal of solvent under reduced pressure.

Synthetic coumarins **1**–**3** were prepared and characterized as we previously reported [31].

### 4.2. Bioassays on Weeds

#### 4.2.1. Seed Germination Bioassay

The potential phytotoxic activity of the three synthetic coumarin derivatives (**1**–**3**) was assayed on seed germination of two noxious weeds: *Amaranthus retroflexus* and *Echinochloa crus-galli*. These species has been chosen not only for their economic importance [71,72] but also because they are representatives of the two different classes Liliopsida and Magnoliopsida. For the experiments, all the molecules were firstly dissolved in 0.1% EtOH (*v*/*v*) and then diluted in deionized water to reach the final concentrations: 0, 50, 100, 200, 400, and 800 µM. The same amount of ethanol employed to solubilize the molecules was added in the control.

Seeds were surface sterilized for 15 min using a 15% (*v*/*v*) NaClO solution and then rinsed three times with sterile deionized water. Ten seeds for each species were distributed into a Petri dish (6 cm diameter) on a double layer of filter paper moistened with 2 mL of each concentration and molecule. Control treatments received 2 mL of distilled water. Petri dishes were then placed in a growth chamber at 25 ± 1 °C, 70% relative humidity, in dark conditions. Seeds germination was recorded every 24 h for five consecutive days. The total germination index [G_T_ (%)], germination speed (S), and speed of accumulated germination (AS) were calculated as reported by Chiapusio *et al.* [73]. At the end of treatments, the un-germinated seeds were carefully washed, placed in Petri dishes containing filter paper, wetted with deionized water and checked daily for three days in order to evaluate the reversible or irreversible inhibition of the seed germination process [74].

#### 4.2.2. Root Elongation Bioassay

Five pre-germinated seeds of *A. retroflexus* and *E. crus-galli*, selected for uniformity in root length (1 mm), were placed in Petri dishes (6 cm diameter) on a double layer of filter paper moistened with 2 mL of the synthetic coumarin solutions at the same concentrations applied for seed germination bioassay (Section 2.2.1). Petri dishes were then placed in a growth chamber at 25 ± 1 °C and 70% relative humidity. After 48 h of exposure, Total Root Length (TRL) was measured according to Araniti *et al.* [75].

### 4.3. Bioassays of Arabidopsis thaliana

#### 4.3.1. Seedling Growth Bioassays

*Arabidopsis thaliana* (L.) Heynh. seeds ecotype Columbia (Col-0) were surface sterilized for 3 min in 50% EtOH and NaOCl 0.5% with Triton X-100 at 0.01% and then rinsed three times in distilled water. After sterilization, seeds were maintained in 0.1% agar at 4 °C for 72 h to promote the synchronization of germination. Then, 24 sterilized seeds were sown in Petri dishes (100 × 150 mm) containing agar medium (0.8% *w*/*v*) enriched with micro- and macronutrients (Murashige-Skoog, Sigma-Aldrich) and supplemented with 1% sucrose. According to Araniti *et al.* [76] plates were placed vertically in the growth chamber (22 ± 2 °C temperature and 75 mol·m^−2^·s^−1^ light intensity) to promote geotropic root growth.

Once germinated, 24 seedlings (7 day old) were transferred to a single plate and grown for 14 days in the same medium containing the synthetic coumarin derivatives at the same concentrations previously reported (Section 2.2.1). All the molecules were previously dissolved in 0.1% EtOH (*v*/*v*), and then autoclaved. The same amount of EtOH was added to the control plates.

After 14 days of treatments, seedlings were collected and separated into shoot and root. Shoot Fresh Weight (SFW) and Leaf Number (LN) were evaluated. Whole root system was imaged by scanning (STD 1600, Régent Instruments Inc., Quebec, QC, Canada) and Total Root Length (TRL), Number of Lateral Roots (NLR) and Lateral Root Length (LRL), using WinRhizo Pro system v. 2002a (Instruments Régent Inc., Quebec, Canada) were measured. Root Hair Density (RHD) was analyzed by using a stereoscopic microscopy (Olympus SZX9, Jackson, MS, USA).

#### 4.3.2. Determination of Mitotic Sites

The number of mitotic sites was evaluated as described by Canellas *et al.* [77], with some modifications. After 14 days of treatment with synthetic coumarin derivatives, roots were washed in 50 mM phosphate buffer (pH 7.4) and then warmed at 75 °C in 0.5% KOH (*w*/*v*) solution for 20 min. The roots were carefully rinsed in 50 mM phosphate buffer (pH 7.4) and then stained in haematoxylin solution containing 0.025 g haematoxylin, 0.0125 g ferric ammonium sulphate and 1.5 mL of 45% (*v*/*v*) acetic acid for 16 h, in dark conditions. Finally, the roots were distained in 80% lactic acid (*w*/*v*) at 75 °C for 20 s, and the mitotic sites were counted by stereoscopic microscopy (Olympus SZX9).

#### 4.3.3. Pigment Quantification

Chlorophyll a (Chl_a_), chlorophyll b (Chl_b_), and carotenoids (Ct) quantification was carried out on *A. thaliana* seedlings treated with each molecule for 14 days (see Section 2.1). One hundred milligrams of leaf tissue for each replicate and treatment were ground in liquid nitrogen and extracted with 1.5 mL methanol. The extract was then centrifuged at 170 g for five minutes and, successively, 500 µL of supernatant was mixed with 500 µL methanol. The absorbance of the extracts was determined at 470, 653, 666 and 750 nm. The pigment content (mg·g^−1^ of SFW) was evaluated according to the following Equations proposed by Wellburn [78]:

(1) $${\text{Chl}}_{a}\left(\mu g\right)=\left(15.65\left({\text{DO}}_{666}-{\text{DO}}_{750}\right)-7.34\left({\text{DO}}_{653}-{\text{DO}}_{750}\right)\right)\times V$$

(2) $${\text{Chl}}_{b}\left(\mu g\right)=\left(27.05\left({\text{DO}}_{653}-{\text{DO}}_{750}\right)-11.21\left({\text{DO}}_{666}-{\text{DO}}_{750}\right)\right)\times V$$

(3) $$C_{t}\left(X+C\right)\left(\mu g\right)=\left(1000\left({\text{DO}}_{470}-{\text{DO}}_{750}\right)-2.86{\text{Chl}}_{a}-129.2{\text{Chl}}_{b}\right)/221)\times V$$

where DO is the optical density, V is the volume of methanol used (mL) and (X + C) the sum of carotenoids and xanthophylls. 

### 4.4. Statistical Analysis

A completely random design with 5 replications was adopted to evaluate the effects of synthetic coumarin derivatives on seed germination and root growth of both weeds and seedlings growth of *A. thaliana*. Data were evaluated for normality (Kolmogorov-Smirnov test) and tested for homogeneity of variances (Levene’s test). The statistical significance of differences among group means were estimated by analysis of variance followed by Tukey test in case of homoscedastic data, and by Tamhane’s T2 test in the case of heteroscedastic data (*p* ≤ 0.05). All statistical analyses were performed by using SPSS ver. 6.1 software (Insightful Corporation, Seattle, WA, USA). According to Araniti *et al.* [79] the SFW, TRL, NLR, RHD responses to different doses of all molecules were evaluated by a nonlinear regression model using a log-logistic function that allowed to estimate the ED50 parameter, the dose required to reduce 50% of the total response. The phytotoxicity comparison among the three molecules was performed by one-way ANOVA using the ED_50_ as a variable with the molecule as main factor. The ED_50_ data were first checked for deviations from normality (Kolmogorov-Smirnov test) and tested for homogeneity (Leven Median test). The statistical significance of differences among group means were estimated by analysis of variance followed by Tukey test in case of homoscedastic data, and by Tamhane’s T2 test in the case of heteroscedastic data (*p* ≤ 0.05).

## 5. Conclusions

In conclusion, all the synthetic coumarins **1**–**3** exhibited a strong phytotoxic activity among *Arabidopsis* seedlings affecting shoot and root growth as well as root morphology, seed germination and root growth of two noxious weeds higher than the natural coumarin. These results suggested that the three synthetic coumarins could be promising phytotoxic molecules employable as new natural-like herbicides. In perspective, the potential effectiveness of these coumarin derivatives as herbicides will have to be tested in field conditions, where the soil properties and microbial interactions come into play.
